# Interleukin‐6 receptor inhibitor tocilizumab ameliorates periodontal inflammation in patients with rheumatoid arthritis and periodontitis as well as tumor necrosis factor inhibitors

**DOI:** 10.1002/cre2.11

**Published:** 2015-11-13

**Authors:** Tetsuo Kobayashi, Satoshi Ito, Daisuke Kobayashi, Anri Kojima, Atsushi Shimada, Ichiei Narita, Akira Murasawa, Kiyoshi Nakazono, Hiromasa Yoshie

**Affiliations:** ^1^ Division of Periodontology, Department of Oral Biological Science, Graduate School of Medical and Dental Sciences Niigata University Niigata Japan; ^2^ General Dentistry and Clinical Education Unit Niigata University Medical and Dental Hospital Niigata Japan; ^3^ Niigata Rheumatic Center Shibata Japan; ^4^ Division of Clinical Nephrology and Rheumatology, Department of Homeostatic Regulation Developments, Graduate School of Medical and Dental Sciences Niigata University Niigata Japan

**Keywords:** Arthritis rheumatoid, immunotherapy, interleukin‐6 receptor, periodontitis, tumor necrosis factor‐alpha

## Abstract

Interleukin‐6 (IL‐6) may play a pathological role in rheumatoid arthritis (RA) and periodontitis. Although the efficacy of medication with IL‐6 receptor inhibitor, tocilizumab (TCZ), has been demonstrated in the treatment of RA, very little is known about whether TCZ therapy affects periodontitis. The aim of the present study is to compare periodontal condition in patients with RA and periodontitis before and after TCZ therapy. The study participants consisted of 20 patients with RA and periodontitis who were treated with TCZ and 40 patients with RA and periodontitis who received medication with tumor necrosis factor inhibitor (TNFI). Clinical periodontal and rheumatologic assessments and serum biochemical measurements using enzyme‐linked immunosorbent assays were performed at baseline and 3 and 6 months later. TCZ and TNFI therapies significantly reduced periodontal inflammation that was determined by gingival index, bleeding on probing, and probing depth (*p* < 0.017), although plaque levels were comparable before and after the therapies. Both therapies also significantly decreased disease activity score including 28 joints using C‐reactive protein (CRP), number of tender and swollen joints, and serum levels of anti‐cyclic citrullinated peptide antibodies, rheumatoid factor, CRP, and matrix metalloproteinase‐3 (*p* < 0.017). Additionally, a significant decrease was observed in periodontal clinical attachment level after TCZ therapy (*p* < 0.017), but not after TNFI therapy. TCZ therapy significantly decreased serum levels of TNF‐*α*, total immunoglobulin G, and serum amyloid A (*p* < 0.017), although serum levels of IL‐6 and soluble IL‐6R were significantly increased (*p* < 0.017). These results suggest a beneficial effect of TCZ therapy on levels of periodontal inflammation in patients with RA and periodontitis, which might be related to decrease in serum inflammatory mediators.

## Introduction

Rheumatoid arthritis (RA) is a systemic autoimmune disease characterized by chronic inflammation and joint tissue destruction, which results in functional disability. Periodontitis represents a chronic inflammatory disease that affects tooth‐supporting tissues and is initiated by infection with oral anaerobic bacteria. Both diseases are common chronic inflammatory conditions and share many clinical and pathologic features (Bartold et al., 2005; de Pablo et al., 2009). Patients with RA were more likely to have periodontitis (Pischon et al., 2008; Dissick et al., 2010), whereas patients with moderate‐to‐severe periodontitis had higher prevalence of RA than those without periodontitis (Dissick et al., 2010; Demmer et al., 2011). Additionally, a similar profile of cytokines has been involved in the pathogenesis of RA and periodontitis (Bartold et al., 2005; de Pablo et al., 2009). These observations suggest that certain features of the inflammatory responses are common to both diseases, which might be underpinned by biologic pathways.

Interleukin‐6 (IL‐6) and tumor necrosis factor‐alpha (TNF‐*α*) are pro‐inflammatory cytokines that regulate immune response and bone metabolism and have been suggested as one of the most potent cytokines that were associated with periodontitis and RA (McInnes & Schett, 2007; Irwin & Myrillas, 1988; Nibali et al., 2012; Graves & Cochran, 2003). It has been reported that patients with periodontitis displayed increased levels of IL‐6 and TNF‐*α* in inflamed gingival tissues, gingival crevicular fluids, and sera than the healthy controls (Takahashi et al., 1994; Lee et al., 1995; Tervahartiala et al., 2001; Kurtiş et al., 2005; Shimada et al., 2010). Serum concentrations of IL‐6 were decreased following periodontal treatment in patients with periodontitis (Shimada et al., 2010; D'Aiuto et al., 2004; Vidal et al., 2009). Likewise, patients with RA showed higher levels of IL‐6 and TNF‐*α* in sera, synovial tissues, and synovial fluids than those with non‐inflammatory arthritis (McInnes & Schett, 2007; Wood et al., 1992; Houssiau et al., 1988; Bozkurt et al., 2000). Furthermore, it was found that serum levels of IL‐6 and TNF‐*α* were positively correlated with disease activity of RA (Kobayashi et al., 2010). These findings imply that constitutive overproductions of IL‐6 and TNF‐*α* play a pathological role in periodontitis and RA.

Tumor necrosis factor inhibitor (TNFI) infliximab (IFX: a chimeric mouse/human anti‐TNF‐*α* monoclonal antibody) proved beneficial in suppressing periodontal diseases in patients with RA (Pers et al., 2008; Mayer et al., 2009; Mayer et al., 2013). An improvement of periodontal condition was also observed in the patients who received IFX, etanercept (ETN: a recombinant fusion protein linked to human type II TNF receptor‐Fc portion), or adalimumab (ADA: a humanized anti‐TNF‐*α* monoclonal antibody) (Ortiz et al., 2009; Üstün et al., 2013; Kobayashi et al., 2014). Other TNFI includes golimumab (a humanized anti‐TNF‐*α* monoclonal antibody that was generated and affinity matured in an in vivo system) and certolizumab pegol (a pegylated humanized Fab' fragment of an anti‐TNF monoclonal antibody with a high affinity for TNF‐*α*), both of which have not been studied in relation to periodontitis.

Tocilizumab (TCZ) is a recombinant humanized anti‐human IL‐6 receptor (IL‐6R) monoclonal antibody. It has been documented that TCZ bound to the IL‐6 binding site of human IL‐6R and competitively inhibited IL‐6 signaling that was mediated by two functional membrane proteins: an 80‐kDa ligand‐binding chain, IL‐6R, and a 130‐kDa non‐ligand‐binding signal‐transducing chain (glycoprotein 130 [gp130]) (Nibali et al., 2012; Nishimoto et al., 2008). TCZ displayed therapeutic efficacy on adults with moderate‐to‐severe active RA in the CHARISMA, OPTION, SATORI, and SAMURAI studies (Maini et al., 2006; Smolen et al., 2008; Nishimoto et al., 2009; Nishimoto et al., 2007). Recently, the authors demonstrated that patients with RA and periodontitis during TCZ therapy showed a greater decease in levels of periodontal inflammation than those who received medication without TCZ (Kobayashi et al., 2014). However, these results were obtained from the comparison between the patients who had been treated with TCZ for approximately 20 months and the patients who had never received TCZ therapy, and there has been no study that evaluated periodontal condition before and after TCZ therapy. Additionally, little is known about the effects of TCZ therapy on soluble receptors for IL‐6, acute‐phase proteins, and immunity that is mediated by macrophages, T cells, and B cells in patients with RA and periodontitis.

Therefore, the aim of the present study is to compare periodontal parameter values and serum levels of IL‐6, sIL‐6R, soluble gp130 (sgp130), serum amyloid A (SAA), TNF‐*α*, IL‐17, and total immunoglobulin G (IgG) in patients with RA and periodontitis before and after TCZ therapy. Furthermore, the effects of treatment with TCZ and TNFI on periodontal condition are compared in the present study.

## Materials and Methods

### Study design

This is a longitudinal case–control study.

### Study participants

**Figure 1:**
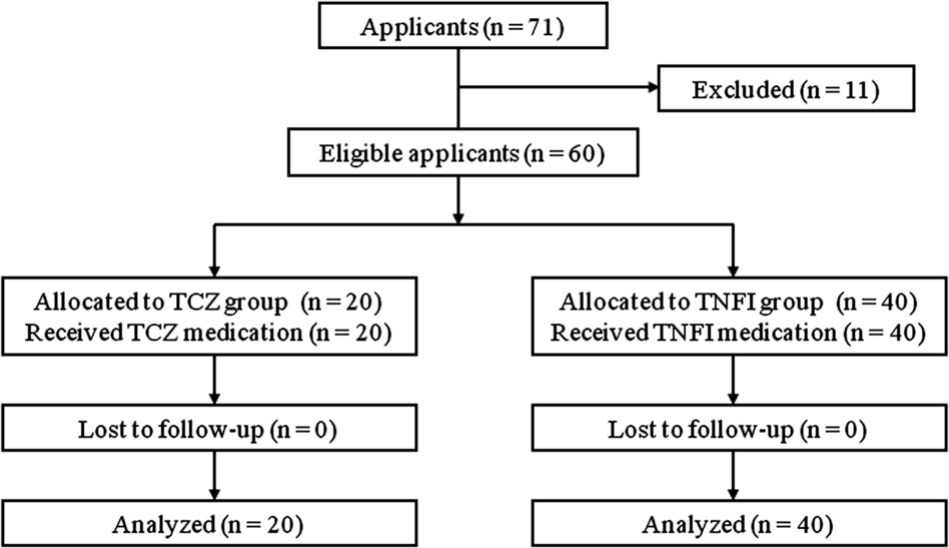
Flowchart of participants through each stage of the clinical study.

Seventy‐one Japanese patients with RA (64 females and seven male; aged 23 to 81 years; mean age: 55.5 years) followed at the Niigata Rheumatic Center, Shibata, Japan, were enrolled in the present study between July 2011 and January 2015. The study protocol was approved by the Institutional Review Board of the Niigata University Faculty of Dentistry (No. 23‐R2‐11‐05, on 12 July 2011) and Niigata Rheumatic Center (No. 2, on 1 June 2011). Signed informed consent was obtained from all participants before their inclusion in the present study. All patients were confirmed to fulfill the 2010 RA classification criteria of American College of Rheumatology and European League Against Rheumatism (EULAR) (Aletaha et al., 2010). Of the 71 patients, 11 were excluded according to the following criteria: diabetes mellitus and pregnancy; antibiotic treatment within the previous 3 months; any periodontal therapy and mouthrinse use within the previous 3 months, fewer than 15 teeth; and absence of periodontitis, as determined by the absence of sites with clinical attachment level (CAL) >3 mm (Esen et al., 2012). Finally, eligible 60 patients with RA were divided into two groups for TCZ or TNFI on the basis of treatment needs by the rheumatologists (S. I., D. K., and A. M.) according to EULAR consensus statement and recommendation (Smolen et al., 2013; Smolen et al., 2014). The flowchart of all participants through each stage of the present study is shown in Figure 1.

The TCZ group consisted of 20 patients with RA (19 females and one male; aged mean ± standard error 54.5 ± 2.6 years) who had taken corticosteroids, disease‐modifying antirheumatic drugs (DMARDs), and non‐steroidal anti‐inflammatory drugs (NSAIDs). Of 20 patients in the TCZ group, nine had been treated with TNFI (three cases of IFX, one case of IFX + ETN, one case of ADA, one case of ADA + ETN, one case of IFX + ETN + ADA, and two cases of certolizumab pegol) and then switched to medication with TCZ intravenously (8 mg/kg, every 4 weeks for four cases) or subcutaneously (162 mg, every 2 weeks for five cases). Other 11 patients had never been treated with TNFI and were first received TCZ intravenously (eight cases) or subcutaneously (three cases). The TNFI group included other 40 patients with RA (35 females and five males; aged 56.4 ± 1.8 years) who had received corticosteroids, DMARDs, and NSAIDs and then were first treated with TNFI (six cases of IFX, nine cases of ETN, 19 cases of ADA, and six cases of golimumab).

### Clinical assessments

The study schedule consisted of the following steps: (1) clinical and serum/laboratory analyses before the first administration of TCZ or TNFI at baseline and (2) the same analyses after 3 and 6 months of the first administration of TCZ or TNFI. Neither periodontal treatment such as professional scaling and prophylaxis nor tooth‐brushing instruction was performed before or during the study period. Additionally, all participants were instructed not to change their oral hygiene regimens throughout the study period.

Clinical periodontal assessments were performed by one trained and calibrated examiner (T. K.) who was masked from the rheumatologic data and treatment. The calibration was performed before the study with five volunteer subjects in Niigata University Faculty of Dentistry. The reproducibility of the clinical measurements was calculated using the intra‐examiner intraclass correlation coefficient, and a value of 0.90 was obtained for CAL with a difference of ±1 mm. All participants were evaluated clinically in the following measurements: number of teeth present, gingival index (GI) (Löe & Silness, 1963), probing depth (PD), CAL, supragingival plaque accumulation, and bleeding on probing (BOP). The presence or absence of supragingival plaque and BOP were recorded at four and six sites around each tooth, and GI score was measured at four sites, respectively. Measurements of PD and CAL were conducted with a Williams probe at six sites around each tooth and recorded to the nearest millimeter, and every observation close to 0.5 mm was rounded to the lower whole number. The averaged score for whole‐mouth GI, PD, CAL, and the number of sites with supragingival plaque and BOP divided by the total number of sites per mouth and multiplied by 100 were calculated for each subject.

The disease activity of RA was determined with Disease Activity Score including 28 joints using C‐reactive protein (DAS28‐CRP), which was calculated with a formula that took into account the tender and swollen joint counts, the patient's general assessment of their condition scored on a visual analog scale (VAS), and CRP (Inoue et al., 2007). DAS28‐CRP constituted four categories: remission (DAS28‐CRP < 2.3), low (2.3 ≤ DAS28‐CRP < 2.7), moderate (2.7 ≤ DAS28‐CRP < 4.1), and high disease activity (4.1 ≤ DAS28‐CRP), which underestimated disease activity compared with DAS28 using erythrocyte sedimentation rate in Japan (Inoue et al., 2007). Smoking status of the participants was classified as current smokers, former smokers, or never smokers, according to information provided on a standard questionnaire.

### Serum biochemical measurements

Peripheral venous blood samples were obtained by venipuncture from all participants after the clinical assessments. Serum was isolated from the blood by centrifugation at 1500 × g for 20 min and stored at −70°C until used. Serum concentrations of rheumatoid factor (RF) and high‐sensitive CRP, and total IgG were determined with a latex particle‐enhanced and a simple nephelometric method (SRL, Tokyo, Japan). Serum levels of anti‐cyclic citrullinated peptide (CCP) antibodies and those of matrix metalloproteinase‐3 (MMP‐3), IL‐6, sIL‐6R, sgp130, TNF‐*α*, IL‐17, and SAA were determined using sensitive enzyme‐linked immunosorbent assay (ELISA) with the commercially available kits (anti‐CCP antibodies: Medical & Biological Laboratories, Aichi, Japan; MMP‐3, IL‐6, sIL‐6R, sgp130, TNF‐*α*, and IL‐17: Quantikine ELISA kit, R&D Systems, Minneapolis, MN, USA; SAA: LZ test SAA, Eiken Chemical, Tokyo, Japan), according to the manufacturer's instructions. The microtiter plates were read at a wavelength of 450 nm for anti‐CCP antibodies, MMP‐3, sIL‐6R, sgp130, IL‐17, 490 nm for IL‐6 and TNF‐*α*, or 570 nm for SAA using an automated microplate reader (Bio‐Rad Japan Laboratories, Tokyo, Japan). The lower limits of detection for these measurements were as follows: RF, 1.25 IU/mL; CRP, 0.004 mg/dL; total IgG, 0.024 mg/dL; anti‐CCP antibodies, 0.4 U/mL; MMP‐3, 0.009 ng/mL; IL‐6, 0.016 pg/mL; sIL‐6R, 1.3 ng/mL; sgp130, 25 ng/mL; TNF‐*α*, 0.038 pg/mL; IL‐17, 15 pg/mL; and SAA, 8 µg/mL. Positivity of RF and anti‐CCP antibodies was defined as showing more than 15 IU/mL and 4.5 U/mL, respectively. Measurement levels below the lower limit of detection were recorded as being not determined and were deleted from the statistical analyses.

### Statistical analyses

Sample size calculation was performed with a parametric test using the statistical software (IBM spss SamplePower version 3.0, IMB, Chicago, IL, USA) on percent sites with PD ≥4 mm (Kobayashi et al., 2014) before analysis, and the results revealed that more than 12 patients in each of the two groups would exceed 0.8 statistical power with the following assumptions: 5% of *α* level and 0.8 of anticipated effect size. After evaluating the normality of distribution by Kolmogorov–Smirnov tests, differences in parameter values at baseline between the two groups were assessed by Mann–Whitney *U*‐tests, where statistical significance was accepted at *p* < 0.05. Differences in parameter values between the baseline and the observed time point (3 and 6 months later) were evaluated by Friedman and Wilcoxon signed rank tests, where significant differences were detected at *p* < 0.017 with Bonferroni correction.

## Results

### Comparison between the groups at baseline

No significant differences were observed at baseline between the TCZ and TNFI groups in any demographic, periodontal, and rheumatologic parameter values (*p* > 0.05) (Table 1). RA medication including corticosteroids, DMARDs, and NSAIDs was not significantly different in the distribution at baseline between the groups (*p* > 0.05) (Table 1).

**Table 1:** Demographic, periodontal, and rheumatologic characteristics of patients with RA at baseline.

Characteristics	TCZ group (*n* = 20)	TNFI group (*n* = 40)	*p*‐value*
Demographic			
Age (years; mean ± SD)	54.5 ± 11.8	56.4 ± 11.3	0.73
Female (*n* [%])	19 (95.0)	35 (87.5)	0.37
Smoker of current/former/never (*n*)	0/1/19	0/4/36	0.51
Periodontal			
Number of teeth present (mean ± SD)	25.3 ± 4.9	24.9 ± 3.7	0.41
GI (mean ± SD)	1.01 ± 0.12	0.93 ± 0.21	0.05
% sites with plaque (mean ± SD)	37.4 ± 25.8	35.6 ± 18.7	0.78
% sites with BOP (mean ± SD)	8.2 ± 10.2	10.4 ± 11.3	0.16
PD (mm; mean ± SD)	2.57 ± 0.32	2.65 ± 0.34	0.54
% sites with PD ≥4 mm (mean ± SD)	9.9 ± 14.6	11.6 ± 12.1	0.27
CAL (mm; mean ± SD)	2.63 ± 0.31	2.72 ± 0.35	0.47
% sites with CAL ≥4 mm (mean ± SD)	11.5 ± 14.5	13.1 ± 13.0	0.57
Rheumatologic			
Duration of RA (months; mean ± SD)	75.3 ± 74.4	79.6 ± 77.1	0.93
DAS28‐CRP (mean ± SD)	3.93 ± 1.17	3.92 ± 1.14	0.89
DAS28‐CRP category			
Remission/low/moderate/high activity (*n*)	1 /2/10/7	3 /2/20/15	0.82
Corticosteroids (*n* [%])	13 (65.0)	27 (67.5)	0.85
DMARDs (*n* [%])	17 (85.0)	38 (95.0)	0.19
NSAIDs (*n* [%])	8 (40.0)	15 (37.5)	0.85
Serum anti‐CCP titer (U/mL; mean ± SD)	154.2 ± 172.0	124.8 ± 136.5	0.85
Anti‐CCP antibody positive (*n* [%])	14 (70.0)	36 (90.0)	0.05
Serum RF levels (IU/mL; mean ± SD)	212.8 ± 447.5	121.1 ± 166.7	0.71
RF positive (*n* [%])	17 (85.0)	36 (90.0)	0.57
Serum CRP levels (mg/dL; mean ± SD)	2.73 ± 2.78	2.26 ± 2.57	0.62
Serum MMP‐3 levels (ng/mL; mean ± SD)	291.5 ± 301.1	234.4 ± 186.7	0.99

RA, rheumatoid arthritis; TCZ, tocilizumab; TNFI, tumor necrosis factor inhibitor; SD, standard deviation; *n*, number; GI, gingival index; BOP, bleeding on probing; PD, probing depth; CAL, clinical attachment level; DAS28‐CRP, disease activity score including 28 joints using C‐reactive protein; DMARD, disease‐modifying antirheumatic drugs; NSAIDs, non‐steroidal anti‐inflammatory drugs; CCP, cyclic citrullinated peptide; RF, rheumatoid factor; MMP, matrix metalloproteinase.

* The *p*‐value was assessed by Mann–Whitney *U*‐test between the groups.

### Changes in periodontal and rheumatologic parameters after TCZ and TNFI therapies

Both the TCZ and TNFI groups showed a significant decrease in GI, percent sites with BOP, and PD at 3 and 6 months later (*p* < 0.017), although the percent sites with plaque proved comparable before and after the therapies (*p* > 0.017) (Table 2). Additionally, a significant decrease in CAL was observed after TCZ therapy (*p* < 0.017), but not after TNFI therapy (*p* > 0.017) (Table 2). The distributions and changes of deepest periodontal pockets in the TCZ groups were 8 to 5 mm (*n* = 1), 6 to 5 mm (*n* = 1), 5 to 4 mm (*n* = 3), and 4 to 3 mm (*n* = 15). Both groups displayed similar changes in percent sites with PD and CAL ≥ 4 mm after the therapies (*p* > 0.017) (Table 2).

**Table 2:** Periodontal characteristics of patients with RA before and after medication with inhibitors of IL‐6 receptor and tumor necrosis factor.

Characteristics	Group	Baseline	3 months	6 months
GI	TCZ (*n* = 20)	1.01 ± 0.12	**0.85 ± 0.17** *	**0.81 ± 0.18** *
	TNFI (*n* = 40)	0.93 ± 0.21	**0.85 ± 0.22** *	**0.81 ± 0.22** *
% sites with plaque	TCZ (*n* = 20)	37.4 ± 25.8	36.7 ± 23.9	33.2 ± 22.1
	TNFI (*n* = 40)	35.6 ± 18.7	30.7 ± 18.0	28.7 ± 19.2
% sites with BOP	TCZ (*n* = 20)	8.2 ± 10.2	**2.1 ± 3.8** *	**1.9 ± 3.8** *
	TNFI (*n* = 40)	10.4 ± 11.3	**7.1 ± 7.9** *	**6.8 ± 7.6** *
PD (mm)	TCZ (*n* = 20)	2.57 ± 0.32	**2.48 ± 0.22** *	**2.45 ± 0.24** *
	TNFI (*n* = 40)	2.65 ± 0.34	**2.57 ± 0.34** *	**2.51 ± 0.33** *
% sites with PD ≥ 4 mm	TCZ (*n* = 20)	9.9 ± 14.6	4.3 ± 4.5	4.2 ± 4.6
	TNFI (*n* = 40)	11.6 ± 12.1	10.9 ± 14.8	11.5 ± 17.6
CAL (mm)	TCZ (*n* = 20)	2.63 ± 0.31	**2.58 ± 0.30** *	**2.55 ± 0.31** *
	TNFI (*n* = 40)	2.72 ± 0.35	2.73 ± 0.45	2.70 ± 0.44
% sites with CAL ≥ 4 mm	TCZ (*n* = 20)	11.5 ± 14.5	8.8 ± 10.4	8.6 ± 10.4
	TNFI (*n* = 40)	13.1 ± 13.0	15.0 ± 18.0	16.0 ± 20.2

RA, rheumatoid arthritis; GI, gingival index; BOP, bleeding on probing; PD, probing depth; CAL, clinical attachment level; TCZ, tocilizumab; TNFI, tumor necrosis factor inhibitor; *n*, number.

Values represent the mean ± standard deviation, and the bold values show the statistical significance.

* Significantly different from the baseline, as assessed by Friedman and Wilcoxon signed rank tests (*p* < 0.017).

Likewise, both the TCZ and TNFI groups displayed a significant decrease in DAS28‐CRP, the number of tender and swollen joints, VAS, and in serum levels of anti‐CCP antibodies, RF, CRP, and MMP‐3 (*p* < 0.017) at 3 and 6 months later, except for VAS and serum levels of RF after 3 months of TCZ therapy (Table 3). Smoking status and RA medication were unchanged in the distribution from baseline to 6 months later, whereas a trend toward decrease was observed in the dose of medication with corticosteroids, DMARDs, and NSAIDs (data not shown). No adverse events were observed in gingival, oral mucosal, and rheumatologic condition of all participants during the study period.

**Table 3:** Rheumatologic characteristics of patients with RA before and after medication with inhibitors of IL‐6 receptor and tumor necrosis factor.

Characteristics	Group	Baseline	3 months	6 months
DAS28‐CRP	TCZ (*n* = 20)	3.93 ± 1.17	**2.62 ± 0.96** *	**2.18 ± 0.81** *
	TNFI (*n* = 40)	3.92 ± 1.14	**2.56 ± 1.00** *	**2.29 ± 0.77** *
Number of tender joints	TCZ (*n* = 20)	4.90 ± 4.63	**2.85 ± 4.22** *	**1.50 ± 2.09** *
	TNFI (*n* = 40)	5.03 ± 5.64	**1.80 ± 1.92** *	**1.30 ± 1.70** *
Number of swollen joints	TCZ (*n* = 20)	4.20 ± 4.47	**2.40 ± 3.35** *	**1.85 ± 2.68** *
	TNFI (*n* = 40)	4.55 ± 4.65	**2.05 ± 2.92** *	**1.10 ± 1.58** *
VAS (mm)	TCZ (*n* = 20)	42.4 ± 25.8	28.8 ± 23.2	**16.3 ± 11.8** *
	TNFI (*n* = 40)	42.9 ± 19.0	**22.6 ± 16.9** *	**24.4 ± 33.2** *
Serum anti‐CCP titer (U/mL)	TCZ (*n* = 20)	154.2 ± 172.0	**116.3 ± 152.3** *	**117.0 ± 154.5** *
	TNFI (*n* = 40)	124.8 ± 136.5	**102.9 ± 132.1** *	**108.5 ± 145.8** *
Serum RF levels (IU/mL)	TCZ (*n* = 20)	212.8 ± 447.5	187.0 ± 451.5	**96.6 ± 135.8** *
	TNFI (*n* = 40)	121.1 ± 166.7	**82.6 ± 124.6** *	**83.6 ± 167.0** *
Serum CRP levels (mg/dL)	TCZ (*n* = 20)	2.73 ± 2.78	**0.20 ± 0.67** *	**0.03 ± 0.13** *
	TNFI (*n* = 40)	2.26 ± 2.57	**0.61 ± 1.30** *	**0.39 ± 0.69** *
Serum MMP‐3 levels (ng/mL)	TCZ (*n* = 20)	291.5 ± 301.1	**129.4 ± 153.8** *	**104.2 ± 115.4** *
	TNFI (*n* = 40)	234.4 ± 186.7	**133.6 ± 141.7** *	**111.3 ± 101.1** *

RA, rheumatoid arthritis; DAS28‐CRP, disease activity score including 28 joints using C‐reactive protein; VAS, visual analog scale; CCP, cyclic citrullinated peptide; RF, rheumatoid factor; MMP, matrix metalloproteinase; TCZ, tocilizumab; TNFI, tumor necrosis factor inhibitor; *n*, number.

Values represent the mean ± standard deviation, and the bold values show the statistical significance.

* Significantly different from the baseline, as assessed by Friedman and Wilcoxon signed rank tests (*p* < 0.017).

### Comparison of changes in periodontal parameters between the groups

The TCZ group showed a significant decrease in GI from baseline to 6 months later than the TNFI group (*p* < 0.05) (Table 4). No significant differences were observed between the groups in changes in other periodontal parameter values (*p* > 0.05) (Table 4).

**Table 4:** Changes (Δ) in periodontal parameters of patients with RA who received medication with inhibitors of IL‐6 receptor and tumor necrosis factor.

Parameters	TCZ group (*n* = 20)	TNFI group (*n* = 40)	*p*‐value*
Δ GI	−0.21 ± 0.19	−0.11 ± 0.12	**0.03** *
Δ % sites with plaque	−4.2 ± 20.6	−6.9 ± 16.5	0.81
Δ % sites with BOP	−6.3 ± 8.9	−3.7 ± 9.3	0.32
Δ PD (mm)	−0.12 ± 0.17	−0.14 ± 0.13	0.38
Δ % sites with PD ≥4 mm	−5.8 ± 13.4	−0.1 ± 17.4	0.82
Δ CAL (mm)	−0.07 ± 0.14	−0.02 ± 0.20	0.96
Δ % sites with CAL ≥4 mm	−2.9 ± 14.0	+2.8 ± 17.4	0.90

RA, rheumatoid arthritis; GI, gingival index; BOP, bleeding on probing; PD, probing depth; CAL, clinical attachment level; TCZ, tocilizumab; TNFI, tumor necrosis factor inhibitor; n, number.

Values represent the mean ± standard deviation changes from baseline to 6 months after medication (−: decrease and +: increase), and the bold values show the statistical significance.

* Significantly different between the groups, as assessed by Mann–Whitney *U*‐test (*p* < 0.05).

### Changes in serum biochemical measurements after TCZ therapy

**Figure 2:**
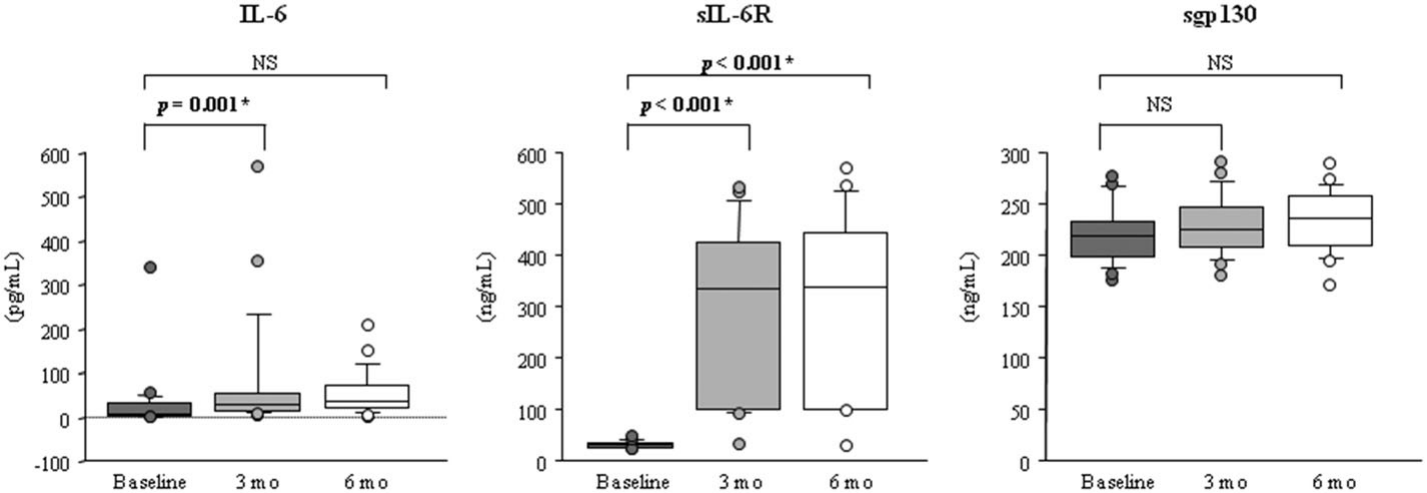
Changes in serum levels of IL‐6, sIL‐6R, and sgp130 after TCZ therapy. The “*” denotes that *p‐*value marks significance of difference between baseline and reassessment (3 and 6 months), as assessed by Friedman and Wilcoxon signed rank tests (*p* < 0.017). IL‐6, interleukin‐6; sIL‐6R, soluble IL‐6 receptor; sgp130, soluble glycoprotein 130; TCZ, tocilizumab; NS, no significant difference.

**Figure 3:**
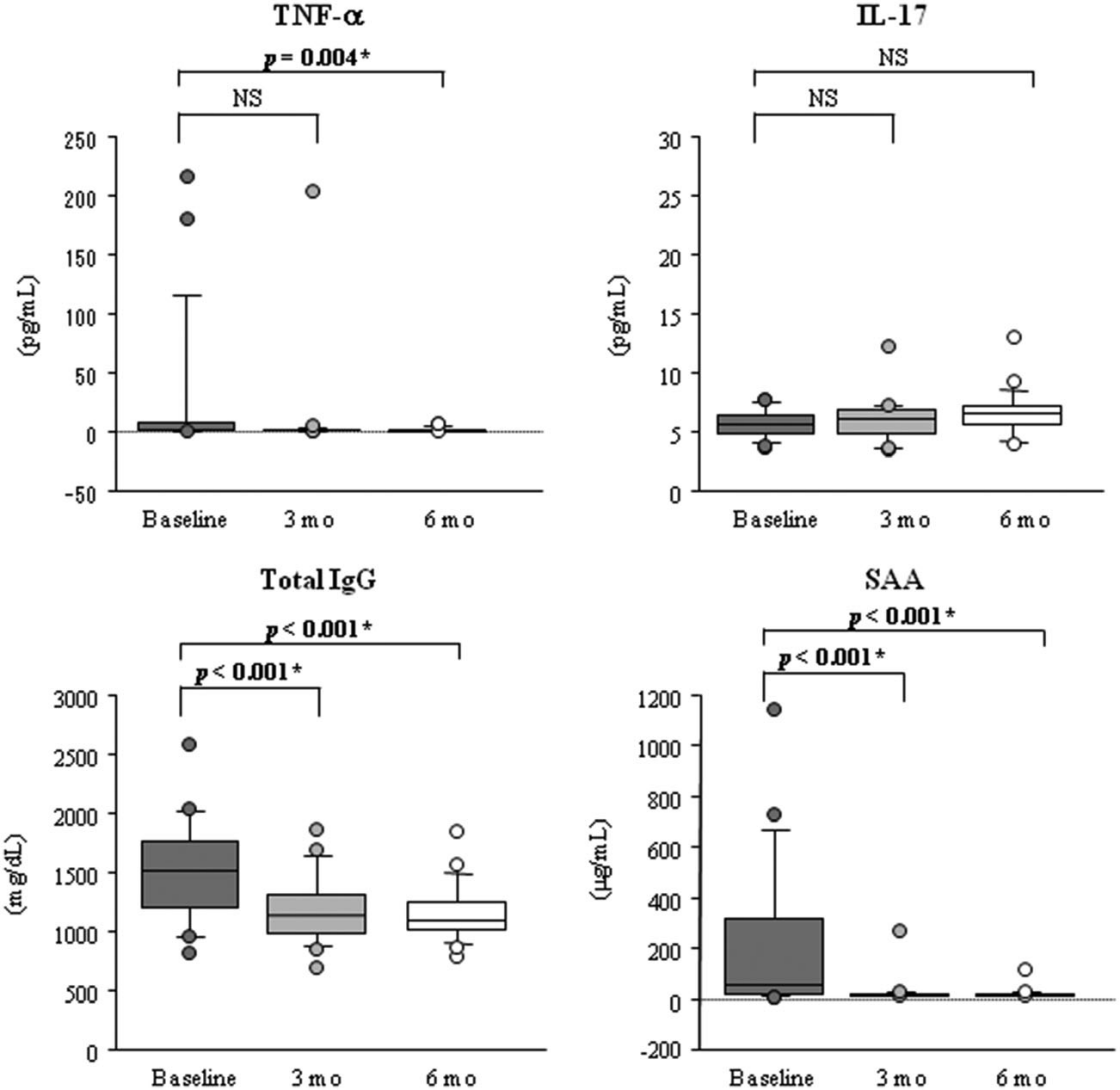
Changes in serum levels of TNF‐*α*, IL‐17, total IgG, and SAA after TCZ therapy. The “*” denotes that *p‐*value marks significance of difference between baseline and reassessment (3 and 6 months), as assessed by Friedman and Wilcoxon signed rank tests (*p* < 0.017). TNF, tumor necrosis factor; IL‐17, interleukin‐17; IgG, immunoglobulin G; SAA, serum amyloid A; TCZ, tocilizumab; NS, no significant difference.

Tocilizumab therapy significantly increased serum levels of IL‐6 at 3 months later (*p* = 0.001) and those of sIL‐6R at 3 and 6 months later (*p* < 0.001 for both comparisons) (Fig. 2). In contrast, TCZ therapy significantly decreased serum levels of TNF‐*α* at 6 months later (*p* = 0.004) and those of total IgG and SAA at 3 and 6 months later (*p* < 0.001 for IgG and SAA at 3 and 6 months later), respectively (Fig. 3). Serum levels of sgp130 and IL‐17 were comparable before and after TCZ therapy (*p* > 0.017) (Figs. 2 and 3).

## Discussion

The results of clinical assessments showed a significant improvement of periodontal inflammation, as demonstrated by decreased averaged levels of GI, BOP, and PD, in patients with RA and periodontitis after medication with TCZ and TNFI. These results are in agreement with the findings of other studies that showed a therapeutic efficacy of TNF inhibition on periodontal condition (Pers et al., 2008; Mayer et al., 2009; Mayer et al., 2013; Ortiz et al., 2009; Üstün et al., 2013; Kobayashi et al., 2014). In particular, TCZ therapy showed a significantly greater decrease in GI than TNFI treatment, which reflects less gingival inflammation in the TCZ‐treated patients. These observations were partially supported by the results of another study that documented periodontitis as a result of excessive host inflammatory responses such as IL‐6 overproduction to microbial challenge (Nibali et al., 2012). Additionally, the clinical data showed a beneficial effect of medication with TCZ, but not with TNFI, on CAL in patients. The improvement of CAL in patients with TCZ might be partially explained by the fact that they had relatively milder levels of periodontitis and might exhibit lower levels of gingival recession after the medication. Moreover, TCZ therapy improved periodontal and systemic inflammation as TNFI although 40% of the patients with TCZ were non‐responder to TNFI, which may underline the therapeutic efficacy of TCZ. With regard to the confounding environmental factor, it was confirmed that the levels of supragingival plaque were comparable and that the status of smoking and RA medication was unchanged during the study period. Therefore, it is suggested that the improvement of periodontal inflammation in the patients who received medication with TCZ and TNFI might be independent of the confounding environmental factors related to periodontal disease. To the best of the authors' knowledge, this is the first study to compare periodontal condition in patients with RA and periodontitis before and after TCZ therapy. However, it cannot be concluded from these findings alone whether TCZ therapy has a beneficial effect on periodontitis, because the limitation of the present study is a relatively small size of patients. In addition, the slight changes in PD between 0.1 and 0.2 mm and in BOP between 4% and 6% after the medication appeared less clinically significant, which might be because the participants did not have very severe periodontitis. In order to confirm and extend the observations obtained from the present study, similar assessments are necessary in a larger number of patients and controls with moderate‐to‐advanced periodontitis. Furthermore, it might be necessary to set the control group that included the patients with RA who only received corticosteroids and NSAIDs. However, it was not permitted ethically that these control patients were monitored for 6 months without any periodontal treatment, because the therapeutic effects of these drugs on periodontitis have not been clarified.

The results of clinical assessments also indicated a significant improvement of rheumatologic condition, as indicated by decrease in DAS28‐CRP, in the number of tender and swollen joints, and in VAS score, in patients after medication with TCZ and TNFI. These findings are consistent with the results of other studies that indicated the therapeutic efficacies of the IL‐6R blockade and TNF inhibition on RA activity and its clinical condition (Maini et al., 2006; Smolen et al., 2008; Nishimoto et al., 2009; Nishimoto et al., 2007; Weinblatt et al., 2003; Atzeni et al., 2006; Potter et al., 2009; Greenberg et al., 2012; Herenius et al., 2013). Additionally, the results showed that TCZ and TNFI therapies significantly decreased serum levels of anti‐CCP antibodies, RF, CRP, and MMP‐3. Moreover, the frequencies of corticosteroids, DMARDs, and NSAIDs medication were unchanged throughout the study period, although these drugs affected systemic inflammatory mediators such as IL‐6 and TNF‐*α* (Barton et al., 1991; Gerards et al., 2003; Renvert et al., 2009). These results suggest that TCZ and TNFI therapies may not only decrease RA activity but also ameliorate systemic inflammation, which may indirectly contribute to the improvement of periodontal inflammation as well. These observations are supported by the results of other studies (Weinblatt et al., 2003; Atzeni et al., 2006; Potter et al., 2009; Greenberg et al., 2012; Herenius et al., 2013; Shimamoto et al., 2013; Nishimoto et al., 2014) that showed the clinical efficacy of TCZ and TNFI therapies to be associated with decrease in serum levels of RF, anti‐CCP antibody, and inflammatory mediators including CRP, pro‐MMP3, MMP‐3, chemerin, and TNF‐*α*. However, it does not rule out the possibility that medication with TCZ and TNFI may play an inhibitory effect on local periodontal inflammation as well, although the levels of TCZ and TNFI have not been studied in the gingival crevicular fluids and periodontium of the patients.

All results in patients with TCZ were analyzed together in the present study, because serum TCZ concentrations were similar between the intravenous and subcutaneous TCZ medications (Ogata et al., 2014). The results of ELISA showed that TCZ therapy significantly increased serum levels of IL‐6 and sIL‐6R, which is consistent with the results of other studies (Nishimoto et al., 2008; Shimamoto et al., 2013). It has been documented that serum levels of IL‐6 depend on the balance between IL‐6 production and clearance and that the increased serum levels of IL‐6 might be partially explained by inhibition of IL‐6R‐mediated IL‐6 clearance from serum because of unavailability of TCZ‐free IL‐6R (Nishimoto et al., 2008). Additionally, the increased serum levels of sIL‐6R might be due to the formation of the sIL‐6R‐TCZ immune complex, because it has been reported that immune complex formation prolongs the elimination half‐life of sIL‐6R in serum (Nishimoto et al., 2008). Nevertheless, TCZ therapy significantly reduced periodontal inflammation and significantly decreased serum levels of TNF‐*α*, SAA, and total IgG, which is in accordance with the results of other studies (Shimamoto et al., 2013; Okuda et al., 2014; Roll et al., 2011). These observations lead to the discrepancy that TCZ therapy may have a beneficial effect on periodontitis, amyloid A amyloidosis, and B cell hyperreactivity, while TCZ medication increased serum levels of IL‐6 and sIL‐6R. A possible explanation for this discrepancy is that IL‐6 trans‐signaling might be completely inhibited by saturation of sIL‐6R with TCZ as long as free TCZ is detectable. Additionally, the results indicated that serum levels of sgp130 proved comparable before and after TCZ therapy, which corresponds to similar levels of healthy individuals that were determined in another study (Narazaki et al., 1993). These findings are supported by the results of other studies that demonstrated unaltered serum levels of sgp130 during inflammation (Narazaki et al., 1993; Müller‐Newen et al., 1998). Although it has been reported that sgp130 interacted with the IL‐6‐sIL‐6R complex to block IL‐6 trans‐signaling (Jostock et al., 2001), it is likely that TCZ therapy did not affect serum levels of sgp130 in patients. Recently, it has been proposed that IL‐6R blockade with TCZ may normalize the activation status of signal transducers and activators of transcription 1 and 3 (STAT1 and STAT3) and may regulate the frequencies of regulatory T cells in relation to reduced inflammation in patients with RA (Ortiz et al., 2015). These points suggest the limitation of the present study that only evaluated serum concentrations of IL‐6, sIL‐6R, and sgp130. Therefore, it is necessary to evaluate the overall changes in the IL‐6R signaling components to clarify the underlying mechanisms through which periodontal inflammation was ameliorated after IL‐6R blockade.

Caution is necessary when the results of periodontal assessments are interpreted, because RA medication may mask any potential effects on periodontal condition. It has been demonstrated that the patients with renal disease who received corticosteroid therapy showed significant lower levels of periodontal inflammation and destruction than the control patients (Tollefsen et al., 1978). However, another study reported that corticosteroid therapy had no influence on periodontal disease (Safkan & Knuuttila, 1984). Additionally, the patients with RA in the treatment with NSAIDs were less likely to have periodontal inflammation and destruction than the controls (Waite et al., 1981), while NSAIDs monotherapy deteriorated GI and CAL (Ng & Bissada, 1998). Moreover, it has been reported that the treatment with methotrexate, a first choice of the DMARDs, exhibited little effects on periodontal inflammatory condition in a rat model of experimental periodontitis (Verzeletti et al., 2007), although its clinical effect has not been studied. Therefore, it is suggested that the effects of corticosteroid, NSAIDs, or DMARDs on periodontitis still remained unclear. Another caution relates to the risk of infections in the patients with RA who received TCZ. It has been reported that TCZ was associated with decreased immune response and higher infection rates in patients with RA (Lang et al., 2012). Therefore, careful monitoring of patients with TCZ should be imposed with respect to infections in the future clinical trials.

In conclusion, the results of the present study suggest a beneficial effect of TCZ therapy on levels of periodontal inflammation in patients with RA and periodontitis, which might be related to decrease in serum inflammatory mediators. Further studies are necessary to determine the beneficial effect of IL‐6R blockade on periodontitis in a larger group of patients and controls.
